# A qualitative study of food sociality in three provinces of South China: social functions of food and dietary behavior

**DOI:** 10.3389/fnut.2023.1058764

**Published:** 2023-10-23

**Authors:** Youxuan Yan, Jindong Ding Petersen, Liuting Lin, Juncai Chen, Xiyi Li, Guiyuan Ji, Fan Zhang

**Affiliations:** ^1^International School of Public Health and One Health, Hainan Medical University, Haikou, Hainan, China; ^2^Hainan Cancer Hospital, Haikou, Hainan, China; ^3^Department of Nutrition and Food Hygiene, School of Public Health and One Health, Guangxi Medical University, Nanning, Guangxi, China; ^4^Guangdong Provincial Institute of Public Health, Guangzhou, Guangdong, China

**Keywords:** South China, food, dietary behavior, social function, qualitative research, sociality

## Abstract

**Background:**

Food sociality refers to the exploration of food production, exchange, distribution, and consumption and influences on cultural communication and social meaning. This study aimed to investigate food sociality in three provinces of South China to provide theoretical and practical evidence of food sociality in the region and to revise nutrition policies.

**Materials and methods:**

We conducted a qualitative study comprising 25 experts in the fields of nutrition, sociology, food science, and agriculture from Hainan, Guangdong, and Guangxi Province by using a semi-structured in-depth interview, which included 28 pre-determined questions covering six topics. The interviews were conducted between November 2020 and March 2021. Verbatim transcripts were analyzed thematically using NVivo 11.0.

**Results:**

Of the 25 experts, the mean age was 50.6 (SD = 8.4) years, 15 (60%) were male, and 22 (88%) held a master's degree or above. The analysis showed that food sociality in three provinces of South China mainly comprises social functions of food and dietary behavior. Regarding social functions of food, the experts expressed that food represents local culture (72%, 18 experts), presents social status and economic power (40%, 10 experts), and is central to special occasions, traditional customs, and etiquette activities (60%, 15 experts). In terms of social functions of dietary behaviors, the majority of experts indicated that food is a social communication tool (72% experts), has geographical characteristics (80% experts), and, to some extent, is used as a proxy for reward or punishment. Furthermore, festivals are one of the core elements of food sociality in the region, although food safety is a major concern. Some dietary behaviors, such as overindulgence in afternoon tea and encouraging drinking, may increase the risk of chronic diseases.

**Conclusion:**

Food sociality in three provinces of South China is mainly related to the social function of food and dietary behavior. It is a combination of local culture, social status and economic power, traditional customs, rewards and punishments, geographical food preference, and social communication tools. The authors recommend increasing food safety at festivals and promoting healthy eating behaviors in order to improve the overall health of the population in this region.

## 1. Introduction

Food sociality refers to the exploration of food production, exchange, distribution, and consumption as a special way to describe cultural communication and communicate social meaning (1). As a behavior with biological and cultural attributes, diet provides an excellent starting point for understanding all social behaviors, concepts, and theories of human groups (2). Though eating is fundamentally a basic physiological need, it is also a symbol and is given many social meanings. This means that the human diet evolves (3). People can understand the meaning of social life and culture of a social network via food, so food sociality is an understanding of the social nature of food.

From ancient times to the present, food has been endowed with expressive and profound cultural connotation by human beings. Under the condition that most people in society can meet their food requirements, human beings not only need food to survive and maintain health, but also to carry out social practices with interactive attributes (4).

Food preferences and consumption can be influenced by economics, politics, culture, and other factors. Food is not only about eating, drinking, nutrition, and digestion, but also about placing the food and eating habits of a regional group in the context of historical movements, thus gaining a unique perspective on the culture of individual and group life (5). Dietary behaviors facilitate “dialogue” between people and food and reflect people's desire to maintain and construct their social orders (6). From an anthropological perspective, food is part of a society that establishes relationships between people, environment, and beliefs (7).

However, many studies exploring food culture in three provinces of South China often overlook the social function of food (8–10). Ma believes that the social functions of food in the world generally include establishing and maintaining interpersonal relationships, expressing the degree of interpersonal relationships, representing social status, explaining group characteristics, celebrating important events, holding symbolic significance, and use as reward or punishment (11), but this study did not focus on South China, and the research on food sociality in the three provinces of South China is still missing. In addition, through reviewing literature, we found that the occurrence of chronic diseases and some special diseases in three provinces of South China may be related to the sociality of their food (12–16). Therefore, this study aimed to investigate food sociality in three provinces of South China, and explain under which conditions and activities the food sociality will emerge, to provide theoretical and practical evidence of food sociality in this region, to reduce the gap between South China and the rest of the world in the interpretation of food culture, and to provide recommended diet-related policies and interventions if necessary.

## 2. Materials and methods

### 2.1. Study design and settings

This qualitative study was conducted in three provinces of South China, namely Guangdong, Guangxi, and Hainan, where the residents' dietary practices and food culture differ significantly from the remaining regions of China. The region has a population of 187 million, a daily average temperature over 20°C, and an annual precipitation of 1,400–2,000 mm, i.e., a hot and rainy, four-season evergreen tropical-subtropical southern zone, with a unique research significance from a geographic perspective (17). Our study focused on the understanding of food sociality of locals by interviewing food-related experts from this region.

The data in this study are qualitative. As an important qualitative research and analysis software, Nivio can enable us to conduct content analysis and research based on Grounded theory, which is applicable to the processing of non-quantitative information such as group discussions, interviews, surveys, videos, audio, and social media. The extraction of files in different formats can be completed through nodes and encoding. Nvivo can significantly improve the quality of research. This software does indeed reduce a large number of manual tasks, giving researchers more time to discover trends, identify themes, and draw conclusions, which is conducive to the rigorous nature of the research and further improves its effectiveness. The analysis of qualitative data has become easier and the results have become more reliable.

### 2.2. Sampling

We used non-random sampling and snowball sampling methods to recruit the study participants, i.e., food-related experts. We identified experts who appeared frequently in academic journals, newspapers, and other authoritative media by using the key words “diet,” “culture,” and “nutrition.” After a pilot study, theme saturation can be achieved by interviewing two experts in each field in each region. Therefore, 24 experts were planned to be recruited, and 25 experts were recruited overall. The inclusive criteria were: ① 10 years or more of working experience in nutrition, sociology, food, or agriculture; ② holding an intermediate or above level of professional or technical title; ③ with a bachelor degree or above; and ④ willing to participate in this study.

### 2.3. Interview

A one-to-one in-depth interview was given to the recruited 25 experts from the nutrition, sociology, and food or agriculture fields.

On the basis of Guansheng Ma's understanding of food sociality, we focused on food sociality from different biological and social perspectives, and discussed the development of the interview outline on this basis, such as the function of food and the meaning of dietary behaviors, putting together specific and subtle activity elements (18). In addition, considering the characteristics of South Chinese residents who like to eat together, we also added the hygiene problem of using serving chopsticks and serving spoons. After a pilot study, the feasibility of the food sociality interview outline was assessed by interviewing three experts from Guangdong, Guangxi, and Hainan, and the interview outline was adjusted according to the test results. Finally, the semi-structured interview outline included 28 pre-determined questions covering six topics (Supplementary Table 1).

The interviews were conducted in Hainan, Guangdong, and Guangxi provinces from November 2020 to March 2021. The total amount of time interviewing 25 experts was 1,322 minutes with an average of 53 minutes (range 47–83 minutes) per interview. Due to the COVID-19 pandemic, three interviews were conducted via video calls using WeChat App; the rest were face-to-face. In the beginning of the interview, the interviewees were asked to describe their experience with food socialization, and then a semi-structured interview started following the pre-determined interview outline. However, during the interview process, the trained interviewers could make certain adjustments according to the interviewee's response. For example, when the interviewees talked about local food, they were asked to describe the significance of the food, and which local festivals the food was associated with.

### 2.4. Data analysis

**Figure 1:**
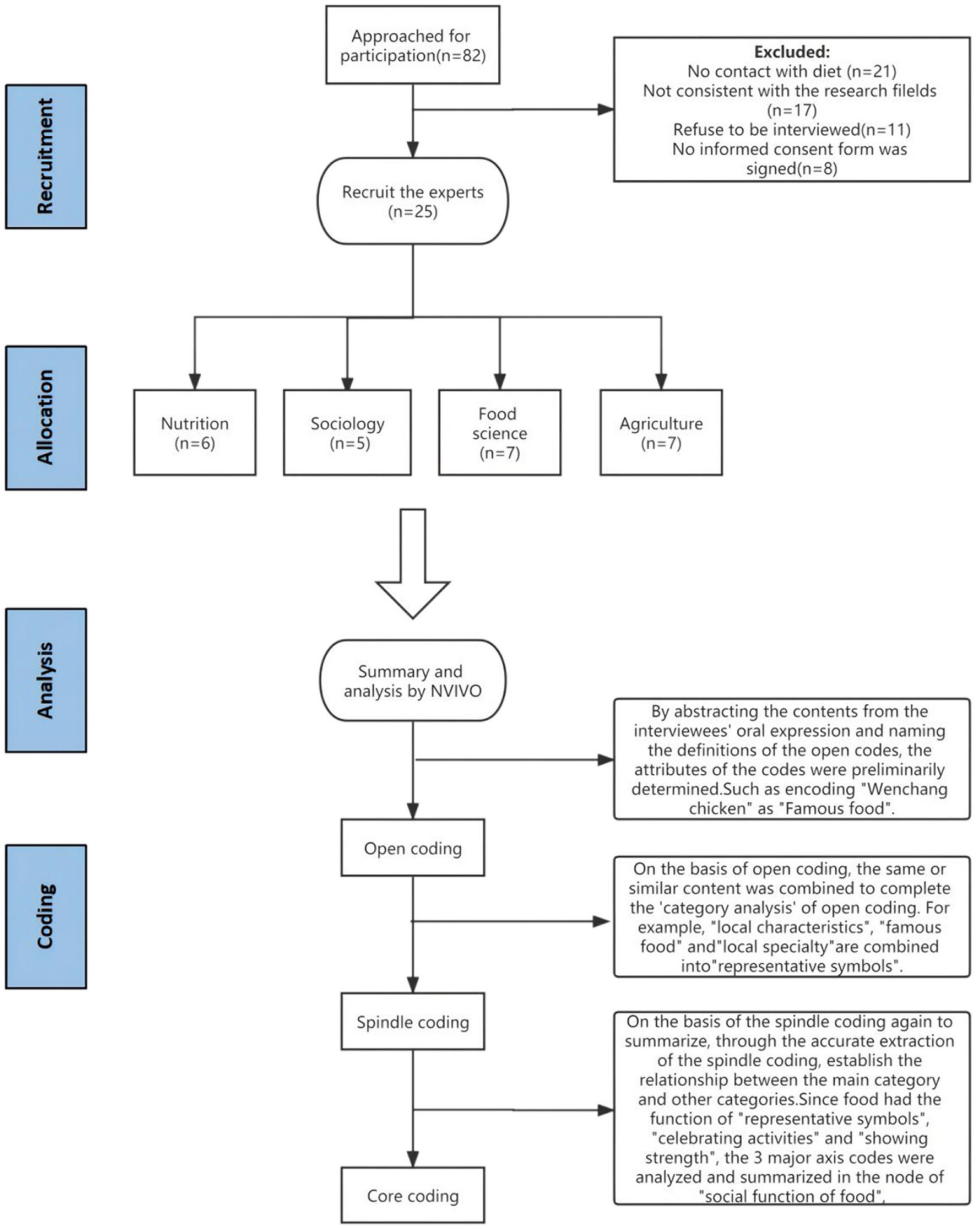
Flowchart of interviewee selection and data analysis for food sociality in three provinces of South China.

A total of 25 interview texts were collected, coded, and summarized for information extraction, node coding, and analysis to summarize the food sociality in three provinces of South China (Figure 1). Three coding stages were used in this study, which were open coding, spindle coding, and core coding.

The first stage was open coding. Based on 25 interviews transcribed, we summarized, re-grouped, re-organized, defined, and coded. A total of 37 open-code nodes were generated. Table 1 illustrates six nodes which showed the most frequent key words mentioned by the interviewees (the rest of nodes are available upon request).

**Table 1:** Open coding (part).

**Open coding**	**Original text**
A1^*^ Famous food	“*The four famous dishes of Hainan: Wenchang Chicken, Jiaji Duck, Hele Crab, and Dongshan Goat. Other dishes included Small Yellow Cattle, Hainan Vegetarian Pot, Salted Dried Fish, Huangliu Old Duck, Hainan Rice Noodles, Lingao Roast Suckling Pig, Shishan Goat Hot Pot, Raw Betel Nut, Coconut Chicken, Coconut Rice, Coconut Boat, Wenchang Chicken rice, and Braised Dongpo Pork.”*
A2^*^ Geographic characteristics	“*If you see someone eating Hainan Rice Noodles and Wenchang Chicken in a foreign country, and loved to drink Afternoon Tea and sit there chatting all day, you will think he comes from Hainan, right? That's our characteristic, right? For example, people in South China are used to ironing their bowls and chopsticks before eating. And people in Guangdong like to drink soup, which is also an obvious feature.”*
A4^*^ Traditional festival food	“*Eating Zongzi is definitely a tradition in the Dragon Boat Festival, and before that there were some customs like drinking Realgar Wine in the north. Then the Mid-autumn Festival would eat Moon Cakes. And we had Glue Pudding for the Lantern Festival.”*
A25^*^ Express the relationship	“*It's also easy to understand that eating with different people have different means. For example, a couple would liked to have a candlelit dinner to express how much they love each other. And he was very particular about dates, such as Chinese Valentine's Day, which needs more attention.”*
A33^*^ Symbol of reunion	“*For people in southern China, we stress social life, morality, emotion, and we eat together with family and friends at the Round Table. Why? Because it means ‘Reunion together'.”*
A34^*^ Light taste	“*Hainan was a coastal area with plenty of seafood. Hainan's local diet is a branch of Cantonese style dish, which is relatively light, and we encourage it to be light now.”*

* A1, main open code 1; A2, main open code 2; A4, main open code 4; A25, main open code 25; A33, main open code 33; A34, main open code 34.

By abstracting the contents from the interviewees' oral expression and naming the definitions of the open codes, the attributes of the codes were preliminary determined. For example, “The four famous dishes of Hainan: Wenchang Chicken, Jiaji Duck, Hele Crab, and Dongshan Goat.” The analysis was summarized as “Famous food,” encoding related content to form 48 reference points. The corresponding Chinese names of the food appearing in the article are shown in Supplementary Table 2.

The second stage was spindle coding, which is also known as relational coding or secondary coding. On the basis of Guansheng Ma's understanding of food sociality and open coding, similar contents were combined for “category analysis” (19). For example, “local characteristics,” “famous food,” and “local specialty” were combined into “representative symbols.” Finally, we identified six spindle codes among 37 open codes based on their similarities and semantic connections, and presented them in Table 2.

**Table 2:** Spindle coding.

**Category**	**Initial concept**
B1^*^ Representative symbols	A1 Famous food, A2 Local specialties, and A3 Local specialties
B2^*^ Celebrating activities	A4 Traditional festival food, A5 Ancestor worship activities, A6 Weddings and funerals, and A7 Emotional sustenance
B3^*^ Showing strength	A8 Economic strength, A9 Curious ingredients, A10 Social status, A11 Rare for expensive, and A12 Show off economic power
B4^*^ Social communication tools	A13 Reach cooperation, A14 Dining together, A15 Establish relations, A16 Drinking Afternoon tea, A17 Contact feelings, A18 Persuade alcohol drinking, A19 Talk about business, A20 Dinner guests, A21 Having Dimsum, A22 Drinking together, A23 Entertainment, A24 Entertain guests, and A25 Express the relationship
B5^*^ Geographic characteristics	A26 Become a habit, A27 Pay attention to atmosphere, A28 Pay attention to emotion, A29 Like to drink soup, A30 Like to eat together, A31 Diet taboo, A32 Original taste, A33 Pay attention to reunion, A34 Light taste, A35 Like fresh ingredients, A16 Drink Afternoon tea, and A21 Having Dimsum
B6^*^ Rewards and punishments measures	A36 As a reward and A37 As a punishment

* B1, main spindle code 1; B2, main spindle code 2; B3, main spindle code 3; B4, main spindle code 4; B5, main spindle code 5.

The third stage was core coding. We established connections between the main and other categories by extracting the spindle coding to define the concept of food sociality in three provinces of South China. Different from Guansheng Ma's study, we focused on the food sociality of the three provinces of South China, and made a new summary according to its characteristics. We believe that food has social significance. After the core coding process, we finally generated two main concepts of food sociality in three provinces of South China: the social function of food and the social function of dietary behavior.

## 3. Results

### 3.1. Demographic characteristics of the interviewees

Table 3 shows the demographic characteristics of 25 interviewees. The mean age was 50.6 (SD = 8.4) years, 15 (60%) were male, and 22 (88%) were experts with a master's degree or above.

**Table 3:** Demographic characteristics of interviewees (*N* = 25).

**Variable**		**Number**	**Percentage**
Gender	Male	15	60.0
	Female	10	40.0
Ethnicity	Han	20	80.0
	Hui	2	8.0
	Li	2	8.0
	Zhuang	1	4.0
Professional field	Nutrition	6	24.0
	Sociology	5	20.0
	Food science	7	28.0
	Agriculture	7	28.0
Education	Bachelor	3	12.0
	Master	12	48.0
	Doctor	10	40.0
Working institute	University	17	68.0
	CDC	3	12.0
	Hospital	2	8.0
	Media	3	12.0
Positional titles	Senior	25	100.0
Province	Guangxi	8	32.0
	Guangdong	10	40.0
	Hainan	7	28.0
Religion	Buddhism	2	8.0
	None	23	92.0

CDC, Center for Disease Control and Prevention.

### 3.2. Social characteristics of food in three provinces of South China

**Figure 2:**
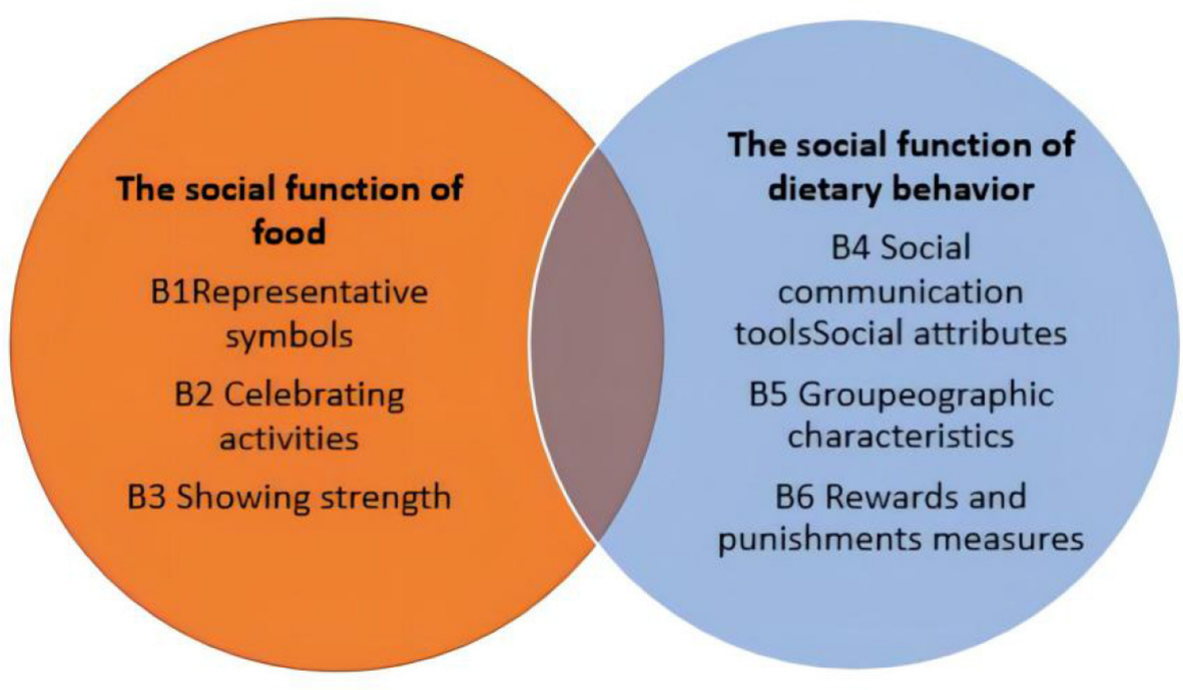
Core codes of food sociality in three provinces of South China.

Based on the analysis and summary of the data from the 25 transcribed texts of the interviews, food sociality in three provinces of South China was found to include two main core codes, six principal codes, and 310 reference points of social function of food and social function of dietary behavior. Core coding results showed the social functions of food are representative symbols, showing strength, and celebration activities. And the social functions of dietary behavior are rewards and punishments, geographic characteristics, and social communication tools. Table 4 and Figure 2 present the content of the food sociality in three provinces of South China both in detail and in overlap, for instance, people communicate with others when they use food for celebrating activities.

**Table 4:** Core coding.

**Main categories**	**Corresponding category**	**Corresponding category specific meaning**	**Material source**	**Reference point**
			***N*** **(%)**	
M1^*^ The social function of food	B1^*^ Representative symbols	Representative food of the three provinces of South China	18 (72)	73
	B2 Celebrating activities	Food prepared for various activities in three provinces of South China	15 (60)	43
	B3 Showing strength	Representative of the strength of food in three provinces of South China	10 (40)	24
M2 The social function of dietary behavior	B4 Social communication tools	Part of the local residents' dietary behaviors could be used as social tools	18 (72)	101
	B5 Geographic characteristics	Some dietary behaviors have become the representative characteristics of local residents	20 (80)	58
	B6 Rewards and punishments measures	Some dietary behaviors could be used as rewards and punishments	7 (28)	11

^*^M1, main core code 1; M2, main core code 2.

^*^B1, main spindle code 1; B2, main spindle code 2; B3, main spindle code 3; B4, main spindle code 4; B5, main spindle code 5.

#### 3.2.1. Social functions of food

There were 140 key points for the social functions of food, including three spindle codes: representative symbols, celebrating activities, and showing strength.

##### 3.2.1.1. Representative symbols

There were 18 (72%) experts who expressed that food was considered to be representative symbols. The most frequently mentioned words were “chicken” and “rice.”

“*Such as Guangdong Rice Rolls, Chaozhou's Casserole Porridge, Hakka Sampan Porridge, Congee with Sweet Potato, Cantonese Style Dumplings. Guangxi's famous Guilin Rice Noodles, Liuzhou River Snails Rice Noodles, Five-color Glutinous rice, Wenchang Chicken rice, Chinese Pudding, Coconut Rice, Bamboo Rice, Hainan Rice Noodles, Danzhou Rice Rotten, Jin Dui, Qingbuliang, Yiba, Hainan Ding An Black Pig Zongzi, and so on*” Guangdong's nutritionist 2 said.

What all of these foods have in common is that they are made of “rice,” which plays an important dietary role in the three provinces of South China, and the residents make full use of local ingredients, all kinds of seafood, poultry, livestock, and vegetables to combine with rice to form its own unique characteristics and become local symbols for the region.

##### 3.2.1.2. Celebrating activities

More than half (15, 60%) of the experts thought that food was used to celebrate activities, such as traditional festivals, ancestor worship activities, weddings, funerals, etc. The most frequently mentioned word was “festival.”

“*People will make many foods that handed down from generation to generation to celebrate the festival. The Three-color Rice, Five-color Rice, and Seven-color Rice of the Li nationality on March The Third. The Mountain Wine, Rice Wine, and Meat Wine that they brewed themselves would be drunk on the festival. During the Spring Festival, there were always allegories. For example, Chickens (pronounced as ‘Ji') are always eaten during the Spring Festival because it means good luck and good wishes (pronounced also as ‘Ji'). Like Celery (pronounced as ‘Qin') means diligent (pronounced also as ‘Qin'), Lettuce (pronounced as ‘sheng') means make money (pronounced also as ‘sheng'), those were very particular because of their pronunciations have particular meanings! Cantonese eat Jishiteng Dessert on the Tomb Sweeping Day. On the morning of the wedding day, you should lay peanuts, Red Dates, Lotus Seeds and rice on your new bed to bless the new couple having babies earlier. You should have New Year Cake and Dumplings for the new year, otherwise there would be no meaning of the Spring Festival. South China's traditional festivals and life rituals are closely related to diet. In traditional festivals, ‘chicken' is homophonic to ‘lucky,' which represents auspicious meaning [sic]*” Hainan's agricultural scientist 2 said.

Festivals are important components of culture, and festivals around the world vary, with food playing a crucial role. The residents in the three provinces of South China appreciate eating food with rich meaning in festivals, such as eating food that would result in good prospects. Various foods have unique meanings due to various histories or pronunciations. Due to this, food is used to celebrate different kinds of activities.

##### 3.2.1.3. Showing strength

Showing strength includes social status and economic power. Unique ingredients are rare and expensive, so using these ingredients demonstrates wealth. Showing strength is one of the social functions of food that was agreed upon by 40% of experts interviewed. The most frequently mentioned word was “precious.”

“*Generally speaking, the harder to get something, the more valuable it is. Just like the Wealthy Shrimp, which used to be cheap and common when the number of shrimps was large, but now the price of shrimp is becoming expensive and it is often eaten at reception parties. If I need to ask somebody for help, I'll take the one to taste precious things to show my sincerity, and maybe the one never eats that food before. It shows that I take the one very seriously [sic]*” Guangxi's sociologist 1 said.

In general, the rarer and more expensive the food provided, the higher the social and economic status of the provider.

#### 3.2.2. Social functions of dietary behaviors

There were 170 reference points on the social functions of dietary behavior. This includes three spindle codes: social communication tools, geographic characteristics, and reward and punishment measures.

##### 3.2.2.1. Social communication tools

In this survey, the majority (72%) of experts suggest that dietary behaviors can be social communication tools. The most frequently mentioned words were “together” and “friends.”

“*The Afternoon Tea and Guangdong Dimsum, Ah, a group of people sitting together to chat, people liked to eat while talking. It's a great way to enjoy food, connect (with each other), exchange information, and even close a deal [sic]*” Guangdong's agricultural scientist 1 said.

“*Intimate Lovers would eat candlelit dinners together, guests were often treated with respect through expensive and rare food, and close friends or colleagues often go to food stalls or restaurants for meals and drinks*” Guangxi's nutritionist 2 said.

In human society, food is used to build and express relationships between people. Dietary behaviors are social. Food consumed by one person alone does not have social properties, but when it is consumed or eaten by a group of people, the sociality of food is determined. Different dining ingredients, environments, and places represent different relationships among diners.

##### 3.2.2.2. Geographic characteristics

After coding and analyzing the group characteristics, a total of 58 reference points were established. Of the experts, 80% believed that food behaviors show geographic characteristics. The most frequently mentioned words were “tea” and “drink.”

“*If you see someone eating Hainan Rice Noodles and Wenchang Chicken in a foreign country, and who loved to drink Afternoon Tea and sit there chatting all day, you will think he comes from Hainan, right? That's our characteristic, right? For example, people in South China are used to heating their bowls and chopsticks before eating. And people in Guangdong like to drink soup, which is also an obvious feature*” Hainan's sociologist 2 said.

“*There is another kind of wine that Guangxi people like to drink. It is called Rice Wine, which is made by ourselves. Self-brewed Rice Wine in many places in Guangxi, including our Liuzhou, Guilin, etc. Rice Wine is also very distinctive. It's actually rare in other provinces, because it's local, cottage and home-brewed [sic]*” Guangxi's food scientist 1 said.

**Figure 3:**
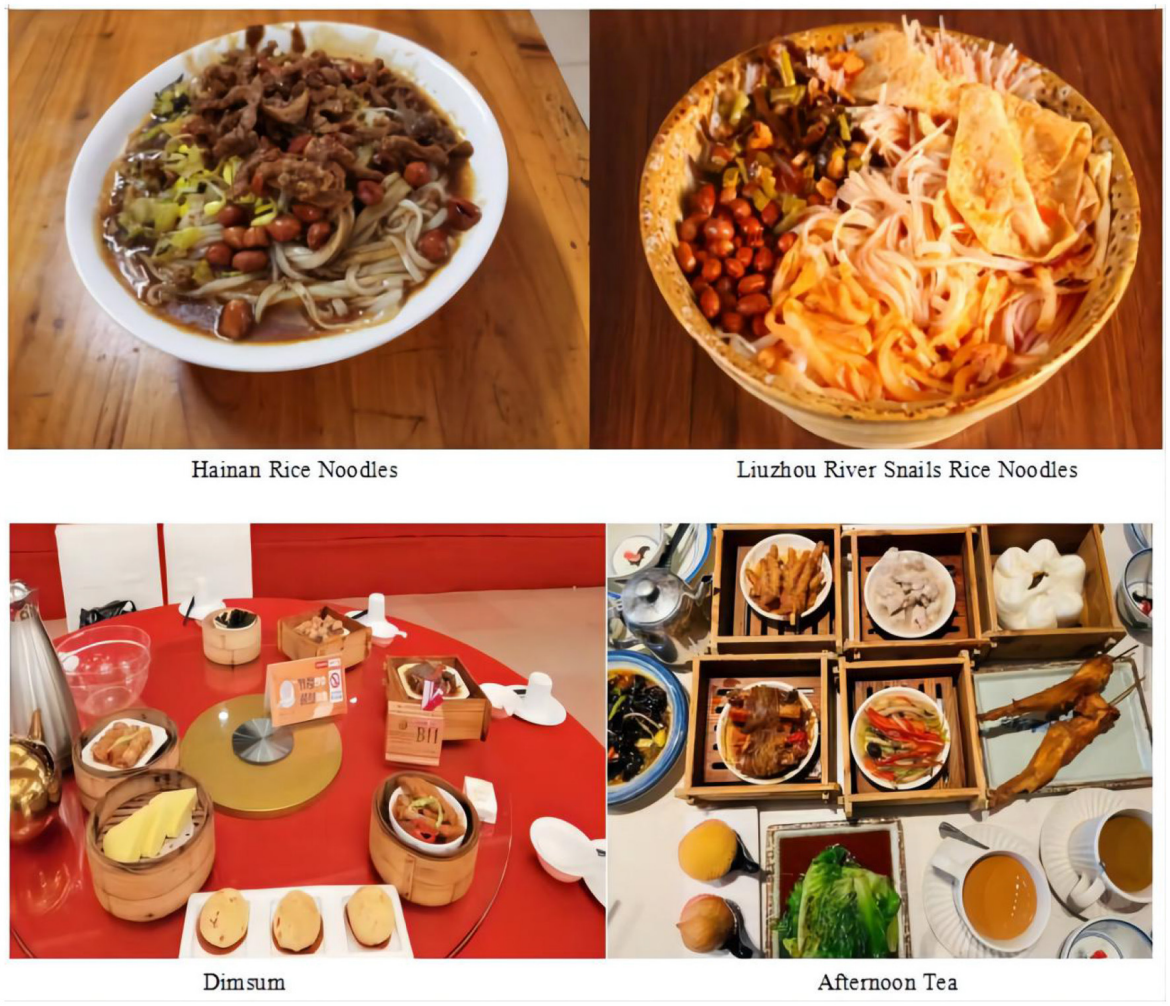
Local symbolic food and dietary behaviors in three provinces of South China (part).

When food becomes a part of the local culture, it remains even after migration. Figure 3 illustrates the role of local symbolic food and dietary behaviors in three provinces of South China. Due to differences in taste and growth environment, food preferences and dietary behavior can be defined as part of geographic characteristics.

##### 3.2.2.3. Reward and punishment measures

Food in this region, in some cases, is used for rewards or punishments (28% experts mentioned), especially to motivate children to behave to meet parents' wishes and expectations. The most frequently mentioned word was “drinking.”

“*For example, when you do well in school, your parents may reward you with candy or promise to take you to a good restaurant. People often use mustard, lemons, and other food as props for punishment when they play games. If you are late for a party, your friends will punish you by drinking wine*” Hainan's sociologist 1 said.

“*In fact, drinking is not all a bad thing. Proper drinking can activate the atmosphere, right? Some people appreciate to drink. Without wine, they are not active in eating. However, on some occasions, some people are unconscious after drinking. It is your fault to persuade them to drink. This phenomenon does exist [sic]*” Guangxi's sociologist 2 said.

To a certain extent, persuading people to drink shows a kind of hospitality. For some people who like wine, drinking is a reward, but when it exceeds a certain standard, it becomes a punishment.

## 4. Discussion

Our study shows that food sociality in three provinces of South China is mainly related to social functions of food and dietary behavior. Social functions of food mainly comprise three aspects: food as a symbol of local culture, food to demonstrate social status and economic power, and food to represent traditional customs and etiquette activities. Social functions of dietary behaviors are expressed by food as a social communication tool, with geographical characteristics, and, to some extent, in use as a proxy for reward or punishment.

Different from Guansheng Ma's understanding, we believe that the social function of food does not represent all characteristics of food sociality, because dietary behavior as a human activity has its social function, while food acts as a bridge between abstract sociality and concrete dietary behavior. In one sense, food has more typical regional characteristics than common dietary behavior. Symbolically, the people in three provinces of South China believed that “chicken” (pronounced as ‘ji') means “lucky” (also pronounced as ‘ji'), lettuce (pronounced as ‘sheng') means to make money (also pronounced as ‘sheng'). The existence of this phenomenon largely depends on the same sound production. They can exist independently of eating behavior. It is a phenomenon that people think of chicken first when they think of lucky. Dietary behavior as a human activity also has its social function; people in the three provinces of South China are used to heating their bowls and chopsticks before eating, so this is an eating behavior with regional characteristics, as people in other places do not have this habit.

Because of the geographical characteristics of the three provinces of South China being rich in rice, where it is the so-called “one land and one people,” rice plays an important dietary role, and the living there make full use of local ingredients to form their own unique dishes that become local symbols (20). Chicken rice, Rice with Chinese Pudding, Coconut Rice, Bamboo Rice and Hainan Rice Noodles from Hainan, Rice Rolls from Guangdong, and Guilin Rice Noodles and Liuzhou River Snail Rice Noodles from Guangxi are the typical local food in three provinces of South China (Figure 3). In northern China, wheat and sorghum are the main food materials, therefore, there are steamed buns, bread rolls, and other steamed or fried wheat food, such as fried hard sticks and fried wheat cakes (21). The dimensions of Japan and three provinces of South China are similar, and Japan is surrounded by the sea, so Japan's fishery has developed very well. Seafood accounts for a large proportion of people's daily diet, so they formed a local culture with “Sushi” as the symbol (22). Each of these local foods is unique and becomes a symbol of region identity, which gives visitors an idea of the local culture.

The development of food is closely related to politics, economy, and culture. It is one of the ways in which society is divided into classes. Rare and expensive food used to be and still is a symbol of social status and wealth (23). This study found that in three provinces of South China, in most situations, people pay more attention to attitude than the price of food. But on some special occasions, such as the occasion where a company or family needs to show their strength, the more expensive the food consumed, the higher the social status the company or family has. As it is still difficult to catch fresh seafood in the deep sea, the price of this kind of food is very expensive in China. The distinctive seafood products are rich shrimp, abalone, etc. This finding is in line with other studies (24, 25). It is the same elsewhere in the world; an English anthropologist, Jack Goody, in his book “*Cooking, Dishes and Class*” describes that differences in food production, distribution, and consumption correspond with differences in social and economic structure. Food distributions are made according to particular classes and ranks (24). A few decades ago in France, food was used as a tool to gain reputation (wealth and social status) by sharing food; other people intended to legitimize their own societal power using food (25).

The social function of food is also reflected in important events such as festivals, weddings, and funerals. In Chinese culture, food is a symbol for the delivery of messages, and is more symbolic than nutritional. This trait can be influenced by different societies and cultures (26). For thousands of years, Northern China has celebrated the Spring Festival by eating dumplings (27), very similar to the tradition of consuming turkey for Thanksgiving in the United States (28). Our study interpreted that in three provinces of South China, the word “chicken” is homophonic to the word “Ji” (lucky), which stands for auspicious meaning, so the character and food appear during the Spring festival, for weddings, Hainan Public Holidays (ancestor worship activities), and other important social occasions, and because of the light taste of the local population, the chicken is generally made into Blanched Chicken. Red Dates, Peanuts, Longan, and Lotus Seeds are laid a couple's bed on the morning of their wedding, which is symbolic of wishing increased fertility and children for the new couple.

Festivals play an important role in food sociality; in Hainan, “Chicken rice” is often used for the “Ancestor Worships” festival, and after the festival, local people gather and share the “Chicken rice” to “*Unite the family ties and connect with the past* (29).” But there is no unified standard for the preservation and manufacture of festival food, and it may become a potential safety hazard. Most festivals are accompanied by banquets, which increases the risk of poor food safety (30). It is recommended to provide better health education during the festival, popularize the knowledge of food production and preservation, formulate corresponding standards, strengthen food hygiene supervision, and prevent food safety accidents.

Moreover, for Chinese immigrants living overseas, food is a symbol of home, especially hometown-food. For instance, some people in Hainan (originally from Wenchang, Hainan) in Malaysia use local Malaysian ingredients to make Wenchang-style “Chicken rice” for “Ancestor Worship.” Anna, who was born and grew up in Macao but currently lives in Portugal, often cooks “Techo,” i.e., a combination of a Portuguese and Macanese dish for the Chinese New Year's Eve and Portuguese Christmas Eve (31).

Dietary behaviors have geographic characteristics. Due to differences in taste and growth environment, food preferences and dietary behavior can be seen as related to geographic characteristics. One's character is shaped by the land they live in and the food they eat. The food in Northeast China is characterized by its “Big serving size.” The Northeast Chinese mostly eat meat and drink alcohol in larger size than people from other regions. Therefore, Northeast Chinese are renowned for their generous, hospitable, easygoing and straightforward attitudes (32). Especially when they host guests, the dish sizes must be more than enough in order to demonstrate the guests are served well. However, in three provinces of South China, gathering together has more meaning than eating. In our research, most experts believe that it is difficult for people in the three provinces of South China to use serving chopsticks and serving spoons. This is related to tradition and habits, which may increase the risk of infectious diseases (33).

In addition, drinking soup and having “Dimsum” and “Afternoon Tea” are popular amongst residents from three provinces of South China and it shows in their geographic characteristics. A study has shown that Dimsum consumers mainly have problems with unbalanced energy intake, high fat intake, insufficient carbohydrate intake, and low intake of vegetables and fruits, all of which increases the risk of chronic diseases such as hypertension and diabetes (34). Therefore, the abundance of Afternoon Tea and Dimsum may be risk factors for chronic diseases. It is suggested that the government should formulate relevant policies to intervene in the moderate intake of high calorie food.

In this study, the experts described how food not only expresses but also builds relationships between eaters. Amongst Cantonese people, having Dimsum is regarded as a way of communicating. People are used to going to tea houses for friends' gathering and business negotiation. While eating, people enjoy the food, connect with each other, exchange information, and even close business deals. Afternoon Tea is also popular in Hainan; as Hainan is located in the tropics, it is hot in the afternoon, the local people are usually used to farming in the morning and evening, and drink Afternoon Tea in the afternoon to rest, known locally as “Dad's Tea.” The sociality of food also existed in the ancient world. The people in western Sudan believe that “*Food can only be shared with relatives and acquaintances, and will never be shared with enemies and strangers* (35).” “*The cultural expression of the public diet becomes an important ethical basis of the community*.” A quotation from the Azander's *sorcery, oracles and magic* reads: “*If a person was not invited to share their food, the absence of that person was often discussed by the neighbors* (36).” “*The sociality of food had become a force to be reckoned with that sustains tradition. Banquet and drinking were the social glue in ancient Greek, they could strengthen civic functions, values, maintain social order, and state stability* (37).” Food or diet alone does not create a social network. Social networks are also developed through special processes, media, and other forms. The ritual and the food create a permanent interaction. Like the “Drinking Ceremony” in ancient Chinese society, “*The Confucian Christian incorporated the idea of respect and caring for the elderly into the village banquets, so that people could be educated when they gathered together for banquets* (38).” Some regard eating as a “Social Act” that is ritualistic. People share food as a communication method and to build connections. It can transcend the meaning of behavior itself and highlight the meaning of other social behavior (39). For instance, through social media such as WeChat (a Chinese social media APP), Twitter, Facebook, or other online platforms, people share delicious food pictures to portray self-satisfaction and build social networks (40).

Food as a means of reward and punishment can be seen all over the world, such as through the use of chocolate or candy (41). Our study found that drinking as a form of punishment and reward were common dietary practices in southern China. Drinking is very common at weddings, workplace dinners, family dinners, and friends' gatherings (42). To a certain extent, encouraging people to drink is a kind of hospitality. For some people who like wine, drinking is a reward, but if it exceeds a certain standard, for example, when it has caused damage to others' bodies, it becomes a punishment. The phenomenon of encouraging people to drink alcohol also appears in other regions of China. What is more distinctive is that northern China tends to drink Erguotou and beer (32), while the residents from three provinces of South China have a habit of brewing their own Rice Wine and sharing it at events. The home-brewed wine contains strong alcohol and causes more damage to different organs through absorption, distribution, metabolism, and excretion than other types of commercial wine (43). We suggest the local government revise policies and implement interventions to promote healthier eating behaviors.

This study summarized the conceptual framework of two dimensions and six aspects of food sociality. Based on insights gained from interviews with the experts, it becomes evident that food is an inseparable element of contemporary human existence. Once food is a part of human activity, its sociality will emerge, so its value is universal and can be used as a reference for other regions to study the local food sociality.

In summary, through this study, we can understand the food sociality in the three provinces of South China, understand under what conditions and activities food sociality will appear, provide theoretical and practical evidence for food sociality, and provide a basis for promoting dietary related policies and intervention measures.

### 4.1. Strengths and limitations

We recruited 25 experts in the fields of nutrition, sociology, food science, and agriculture to increase opportunities for greater participation, which allowed the collection of a wider range of views. To minimize researcher bias in analysis, the third author (LL) checked the coding framework and, importantly, participants were invited to comment on whether findings represented the views shared during interview.

However, this study has limitations. The interview outlines (questions), although tested for their feasibility, were created subjectively without validity. This may lead to selection bias (44).

We only interviewed experts from food-related fields without including local residents with diverse backgrounds. This selection bias may lead to our study being less generalizable; interpreting our results may need caution. In addition, we only included 25 experts from three provinces. Further studies should expand the sample size to increase validity.

## 5. Conclusion

Food sociality in three provinces of South China is a result of the social functions of food and dietary behaviors, which in turn are a result of local culture, social status, economic power, traditional customs, rewards and punishments, geographical food preference, and social communication tools. We suggest strengthening health education, especially for festival food hygiene and healthy eating behaviors, in order to achieve improved population health in this region. Through this study, we have contributed to the summary of food sociality in the three provinces of South China, provided ideas for the summary of food sociality around the world, and provided a basis for modifying dietary policies and intervention measures based on local food sociality in various regions.

## Data availability statement

The original contributions presented in the study are included in the article/Supplementary material, further inquiries can be directed to the corresponding author.

## Ethics statement

The studies involving humans were approved by the Ethics Committee of Hainan Medical University (HYLL-2022-024). The studies were conducted in accordance with the local legislation and institutional requirements. The participants provided their written informed consent to participate in this study.

## Author contributions

FZ initiated the study. JC, LL, XL, and GJ collected and analyzed data. YY drafted the manuscript. JDP revised the manuscript mainly. All authors provided comments for the manuscripts and agreed on the final version.
